# Contrast-Enhanced Computed Tomography Does Not Provide More Information about Sarcopenia than Unenhanced Computed Tomography in Patients with Pancreatic Cancer

**DOI:** 10.1155/2021/5546030

**Published:** 2021-04-23

**Authors:** Yan Zhang, Jiabin Liu, Fei Li, Feng Cao, Ang Li

**Affiliations:** ^1^Department of General Surgery, Xuanwu Hospital, Capital Medical University, Beijing, China; ^2^Department of Radiology, Xuanwu Hospital, Capital Medical University, Beijing, China

## Abstract

**Objective:**

The aim of this study was to understand whether enhanced CT can provide more information than unenhanced CT on diagnosis of sarcopenia.

**Materials and Methods:**

We reviewed the enhanced CT data of 45 patients of pancreatic cancer. Manual tracing of the psoas muscles was used for measuring the cross-sectional muscle areas and attenuation at umbilicus level; afterwards, PMI, PMD, and Δ PMD were calculated.

**Results:**

In the unenhanced scanning, arterial, venous, and parenchymal phases of enhanced CT, PMI values were 6.905 ± 2.170, 6.886 ± 2.195, 6.923 ± 2.239, and 6.866 ± 2.218, respectively, and the difference was not statistically significant. The PMD values at different phases were 34.311 ± 7.535, 37.487 ± 7.118, 40.689 ± 7.116, and 42.989 ± 7.745, respectively, which were gradually increased, and the difference was statistically significant. Meanwhile, the PMD of arterial phase, venous phase, and parenchyma phase showed a linear correlation with PMD of unenhanced scanning phase. 31 patients had low PMD and 14 had normal PMD during the unenhanced scanning phase. With the addition of contrast agent, ΔPMD values increased faster in the low PMD group than in the normal PMD group during the venous and parenchymal phases (7.048 ± 3.067 vs 4.893 ± 2.558; 9.581 ± 3.033 vs 6.679 ± 2.621; *p* < 0.05), which made the gap between PMD after contrast-enhancement *vs.* unenhanced scanning smaller.

**Conclusion:**

The use of contrast agent has no effect on the manually measured PMI values but can change the results of PMD. This change makes the difference of PMD in different enhancement phases smaller than that in plain scan phase and furthermore increases the examination cost; therefore, it is not recommended to use enhanced CT routinely with fixed dose administration of contrast agent for patients' assessment of PMI and PMD.

## 1. Introduction

In recent years, the change of body composition has attracted more and more attention. Body composition, especially muscle wasting, is a debilitating condition that develops with ageing and in various systemic diseases (for example, cancer, renal failure, sepsis, HIV, and trauma) [1]. Muscle wasting is known as sarcopenia. Sarcopenia is a syndrome characterized by progressive and generalized loss of skeletal muscle mass and strength with a risk of adverse outcomes such as physical disability, poor quality of life, and death [2], and the impaired muscular function was more important [3]. Malignancy disease is a major cause of secondary sarcopenia. In cancer patients, sarcopenia is not only related to chemotherapy-induced toxicity [4–6] and the short-term postoperative complications [7], but also closely related to the long-term survival of cancer patients [8–10]; therefore, it is necessary to evaluate the muscle status of cancer patients.

CT is a frequently used evaluation method for the diagnosis, staging, and efficacy judgment of cancer patients; therefore, CT has become a common method for the diagnosis of sarcopenia in cancer patients [11]. In studies using CT as a means of assessing sarcopenia, SMI and SMD are the most commonly used indicators. SMI is the abbreviation for skeletal muscle index, which is calculated as skeletal muscle area divided by the square of the body height [12]. SMI of the L3 lumbar vertebra is found to correspond to the total body skeletal muscle volumes [13, 14] and is the commonly used indicator in clinical study to reflect skeletal muscle mass. SMD is the abbreviation for skeletal muscle density, which is not physical density measured in mg/cm^3^, but muscle attenuation measured in Hounsfield units [15]. SMD was a surrogate measure of muscle quality [16], with reduced HU within skeletal muscle representing increased intramuscular lipid deposition, which has been observed in those with neuromuscular disease [16].

Enhanced CT is increasingly used for the assessment of solid tumors. However, there is no consensus on the assessment of sarcopenia by enhanced CT. In studies of CT in the assessment of sarcopenia, some have used plain CT as the research method [17], some have used enhanced CT as the research method [18], and a considerable number of studies have not reported the types of CT used [19–21]. Enhanced CT can change the attenuation of X-ray due to the introduction of contrast agent, thus changing the SMI and SMD values of patients, but it is not clear whether such a change is more efficient in providing information for the diagnosis of sarcopenia. Therefore, we aimed to compare skeletal muscle mass and density measurements on CT between different contrast-enhancement phases, to understand whether enhanced CT will provide more diagnostic information for sarcopenia than plain CT.

## 2. Materials and Methods

### 2.1. Patients

A total of 45 patients of pancreatic cancer in Xuanwu Hospital of Capital Medical University diagnosed between 2014 and 2019 were selected retrospectively. Inclusion criteria include patients with definite pathological diagnosis, complete clinical data, and available multiphase (unenhanced phase, arterial phase, portal-venous phase, and delayed phase) abdominal CT (CT images were collected prior to any treatment when the patient was present to the hospital). Exclusion criteria include the patients without multiphase abdominal CT examinations. Date of birth, sex, body weight, and body height were collected from the electronic patient medical record within two weeks of the CT examination. Study protocol was approved by the local medical ethical committee.

### 2.2. CT Scanning Protocol

Unified standards were used for the acquisition of CT images in all patients. CT model was Force, Siemens, Germany, daily CT calibration, CT voltage 120kv. Before examination, patients were fasting for 12 hours and were forbidden to drink for 4 hours. The examination was performed in the supine position, and images of unenhanced scan were obtained. Iopromide 100 ml (containing iodine 300 mg/ml) was injected into the right or left antecubital vein, and the contrast regent was injected by bolus injection using a high-pressure syringe at a rate of 3 ml/s. After completion of injection, arterial phase images were obtained by bolus-tracking technique, portal-venous phase acquisition began 30 seconds after arterial phase, and delayed phase acquisition began at 120 seconds after arterial phase. Axial reconstructions were created with a slice thickness of 5 mm in all phases. Images are stored in the PACS system. The images used in this study were electronic copies of CT scans at the level of umbilicus of the abdomen and obtained in the Digital Imaging and Communications in Medicine (DICOM) format from the Hospital Picture Archiving and Communication System (PACS). Data review was performed in a computerized radiology information system (Siemens workstation) used in the radiology department.

### 2.3. Psoas Muscle Mass and Density Measurements: Cutoff Value

**Figure 1:**
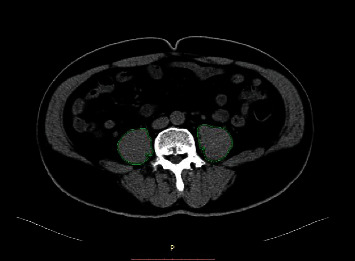
Example of skeletal muscle mass and density measurement on a contrast-enhanced CT slice in the unenhanced scan phase at the level of umbilicus.

In our study, the method of manual measurement of psoas muscle area and density was used. Manual tracing of CT imaging at umbilicus level was used for measuring the cross-sectional areas and attenuation (Hounsfield units [HU]) of the right and left psoas muscles (Figure 1). Data of unenhanced phase, arterial phase, portal-venous phase, and delayed phase were measured, respectively, and being recorded. All measurements were completed by one member of the research team.

#### 2.3.1. Psoas Muscle Mass Index (PMI) Was Calculated by Normalizing the Cross-Sectional Areas for Height (cm2/m2)

PMI= (left psoas muscle areas + right psoas muscle areas)/square of the patient's height [22].

Low PMI using sex-specific cutoff points [22] are 6.36 cm^2^/m^2^ for men and 3.92 cm^2^/m^2^ for women.

#### 2.3.2. Psoas Muscle Density (PMD) Was Calculated by Mean of Left and Right Psoas Muscle Attenuation

PMD= (left psoas muscle attenuation HU + right psoas muscle attenuation HU)/2.

Low PMD was calculated using body mass index- (BMI-) specific cut points [23].

PMD<41 HU for BMI <24.9 kg/m^2^.

PMD<33 HU for BMI >25.0 kg/m^2^.

#### 2.3.3. ΔPMD Was Defined as the Difference between the PMD Values at Different Phases of Enhanced CT and the PMD at Unenhanced Phase

ΔPMD (arterial phase) = PMD (arterial phase) - PMD (unenhanced phase).

ΔPMD (portal-venous phase) = PMD (portal-venous phase) - PMD (unenhanced phase).

ΔPMD (delayed phase) = PMD (delayed phase) - PMD (unenhanced phase).

### 2.4. Statistical Analysis

Data were analyzed using SPSS version 22 (IBM SPSS Statistics, Armonk, New York, USA). One-Sample Kolmogorov-Smirnov Test was used for data normality test. Data were presented as mean ± SD for normal distribution data. Cross sectional surface areas, PMI, and PMD by subphase of CT scan were all compared using analysis of variance for repeated measures. Correlation between PMD by phase of CT scan was conducted using Pearson's correlation coefficient, with linear regression analysis performed to generate regressive equation. The comparison between PMD low group and PMD normal group was made by group data *t*-test. All analyses performed were conducted using two-tailed testing and a *p* value less than 0.05 was considered of significant difference.

## 3. Results

### 3.1. Patient Characteristics

The analytic cohort consisted of 45 patients with pancreas carcinoma, 57.78% of those were male, average age was 61.69 ± 10.36 (39–80), and the patients had an average BMI of 23.56 ± 3.42 (Table 1).

### 3.2. Skeletal Muscle Mass and PMI Measurements

No statistical differences were observed for the average psoas muscle area with CT scan at unenhanced phase, arterial phase, portal-venous phase, and delayed phase (19.328 ± 7.003, 19.280 ± 7.060, 19.384 ± 7.169, and 19.223 ± 7.106 resp.; *p*=0.085). No statistical differences were observed for the PMI value with CT scan at unenhanced phase, arterial phase, venous phase, and delayed phase (6.905 ± 2.170, 6.886 ± 2.195, 6.923 ± 2.239, and 6.866 ± 2.218 resp.; *p*=0.090) (Table 2). The PMI of patients with different enhancement periods in this study is shown in Table 3, the addition of contrast agent did not change the classification of the PMI (normal *vs*. low) in most patients, and there was no significant difference in the proportion of patients with low PMI at different phases.

### 3.3. Skeletal Muscle Density Measurements

**Figure 2:**
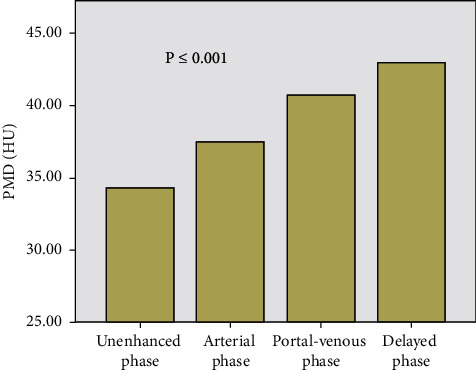
In different phases of enhanced CT, the PMD value gradually increased, and the difference was statistically significant.

An overall difference in PMD was found between the four contrast-enhancement phases (*F* = 188.046, *p* < 0.001). The lowest PMD value was 34.311 ± 7.535 in the unenhanced phase, and the PMD value increased to 37.478 ± 7.118, 40.689 ± 7.116, and 42.989 ± 7.745 in the arterial phase, venous phase, and delayed phase, respectively (Table 4 and Figure 2). The difference between the groups was statistically significant (*p* < 0.001).

We compared the PMD values of different phases in pairs. The results showed that PMD in unenhanced phase was significantly lower than that in arterial, venous, and delayed phases (*p* < 0.001), and PMD in the arterial phase was lower than that in portal-venous and delayed phase (*p* < 0.001).

**Figure 3:**
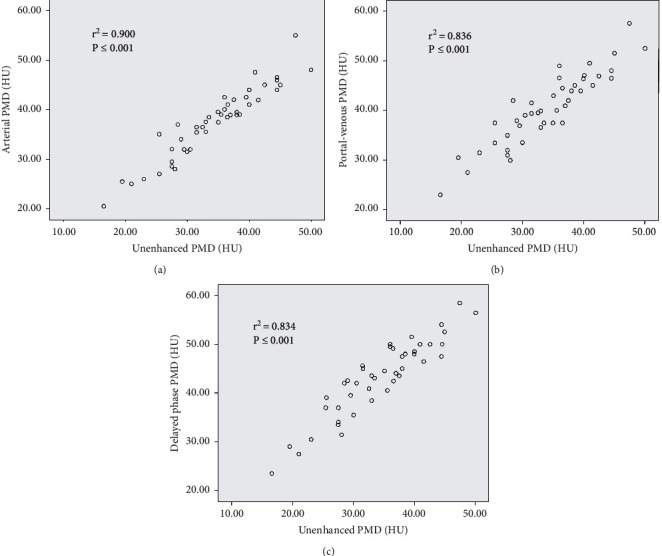
Correlation in SMD values by CT scan phases analyzed (scatter diagram): (a) unenhanced phase *vs.* arterial phase, (b) unenhanced phase *vs.* portal-venous phase, and (c) unenhanced phase *vs.* delayed phase.

The PMD at different phases of enhanced CT was linearly correlated with the PMD value of unenhanced CT : As can be seen by scatter diagram of PMD analysis, i.e., there was a significant positive correlation between unenhanced PMD and other phases of PMD (Figure 3). When the PMD values measured in the different phases were correlated, a significant positive correlation was noted between unenhanced phase *vs.* arterial phase (*r*^2^ = 0.900; *p* < 0.001), unenhanced phase *vs.* portal-venous phase (*r*^2^ = 0.836; *p* < 0.001), and arterial phase *vs.* portal-venous phase scans (*r*^2^ = 0.834; *p* < 0.001).

Linear regression equations calculated from these correlations between the four scan phases are as follows:

Arterial PMD = 6.732 + 0.896*∗* unenhanced PMD.

Portal-venous PMD = 11.065 + 0.863*∗* unenhanced PMD.

Delayed phase PMD = 10.776 + 0.939*∗* unenhanced PMD.

**Figure 4:**
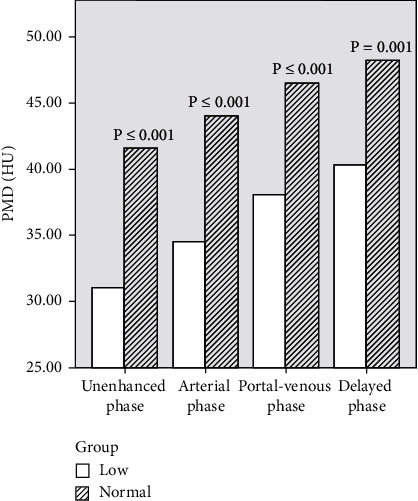
Comparison of PMD values between PMD low group and PMD normal group at different phases of CT : The PMD values of the two groups were lower in PMD low group at different phases of CT, and the differences were significant.

**Figure 5:**
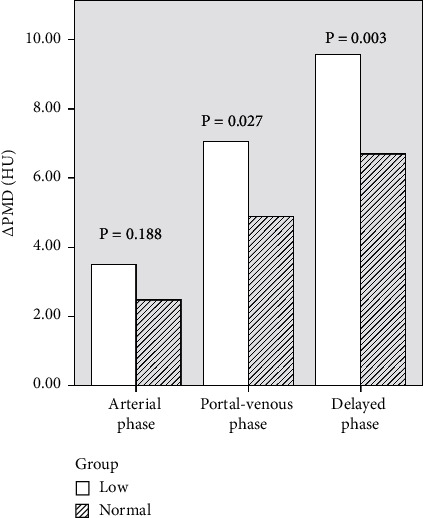
Differences in ΔPMD using contrast-enhancement CT : Regardless of the phase of enhanced CT, in PMD low group, ΔPMD was higher than in PMD normal group, indicating that patients with low PMD had a faster increase in PMD values after enhancement.

During the unenhanced phase, 14 patients had normal PMD and 31 patients had low PMD. In the two groups, the PMD value was lower in the PMD low group at different CT phases, and the difference was significant, *p* < 0.05 (refer to Table 5 and Figure 4). The difference of the mean values of PMD in the two groups was 10.539 in unenhanced phase, 9.520 in arterial phase, 8.383 in venous phase, and 7.637 in parenchymal phase, and the largest difference in PMD could be seen in unenhanced phase. To explore the reasons for the phenomenon, we further examined ΔPMD: we found that no matter in arterial phase, venous phase, and delayed phase of contrast-enhanced CT, patients of PMD low group had higher ΔPMD after enhancement, and patients of PMD normal group had lower ΔPMD after enhancement, and the difference was statistically significant in venous phase and delayed phase (Table 6). In other words, patients with low PMD had a rapid increase in PMD values after enhancement, which resulted in a smaller difference in PMD than in unenhanced phase during each phases of enhancement, and Figure 5 reflects the characteristics. In order to investigate the reasons for the difference in ΔPMD, we further compared the differences in body weight, BMI, PMI, and other related indicators between normal PMD and low PMD group and found that low PMD group, in terms of body weight and psoas muscle area, were lower than normal PMD group (see Table 7 for the results).

## 4. Discussion

To the best of our knowledge, this is the first study to assess sarcopenia by manual measurement of the area and attenuation of the psoas muscle at the level of umbilicus with enhanced CT. Other similar studies have used all muscles of the third lumbar cross section as subjects, and muscle area and attenuation were measured automatically using commercially available software [24, 25]. We used psoas muscle area for the following reasons: psoas muscle area was correlated with lumbar vertebral L3 cross-sectional muscle area, while lumbar vertebral L3 cross-sectional muscle area was positively correlated with whole-body muscle [26]. We used manual tracing of muscles because unless the disease is a lesion of muscle itself, the information of muscle area and attenuation is often not contained in the CT report, while the value of muscle area in the third lumbar vertebral cross section is difficult to obtain manually and should be provided with help from software. If this information is needed in clinical practice, it should be particularly communicated with the radiologist. Manual measurement of the psoas muscle can be done by clinicians themselves and is very convenient; therefore, our study adopted the psoas muscle at the level of umbilicus as the research object and manual measurement as the research method.

Firstly, we analyzed the difference of PMI in different phases of enhanced CT. We used PMI to assess the patients' muscle mass. Skeletal muscle with low PMI is indicative of skeletal muscle mass depletion, also known as sarcopenia. Since the total muscle mass varies according to the race of Asians and Europeans, and the total muscle mass of Asians is lower [27–29], therefore, the normal value in this study used the research data of Japanese which is the same Asian origin as the normal reference value [22]. This study showed that in patients with pancreatic cancer, 17.78% of patients had low PMI, indicating that pancreatic cancer is a wasting disease and that patients with sarcopenia should be concerned. For the effect of enhanced CT on psoas muscle area and PMI, our study showed negative results, which were the same as those of Katie *E* [24], but different from those of Jeroen et al. [25]. The reason why it is different from the study conclusion of Jeroen et al. [25] is that, in our study, the area was measured manually, and the manual measurement depended on the understanding of anatomy and did not rely on the effect of density change, while in the study of Jeroen et al., the software measured automatically, which defined the tissue according to different CT values. When the muscle was enhanced, the CT value would change and thus affect the measurement results. Therefore, our study showed that the anatomy-based manual measurement was not interfered by the contrast agent, suggesting that if the manual measurement method is used, the PMI data obtained at different phases of enhanced CT can be universal; therefore, the addition of contrast agent does not bring more information.

In our study, not only the effect of contrast agent on PMI but also the effect on PMD was assessed. Low PMD means skeletal muscle with low radiodensity, which is suggestive of myosteatosis (intramuscular fatty infiltration), representing impaired muscle function. Our study showed that PMD values were different in patients with different phases of enhanced CT. PMD was the lowest in unenhanced phase, began to increase in arterial phase, and further increased from venous phase to parenchymal phase, and the increase in PMD at different enhancement phases was linearly correlated with the patient's initial PMD, which is consistent with Katie et al. [24] and Michael et al. [30] studies. The reason for this phenomenon is that the attenuation in the blood vessels is increased after the contrast agent enters the tissue blood vessels, thereby increasing the PMD of the measured tissue. With the gradual increase of the concentration of contrast agent in the tissue of the arterial phase, venous phase, and parenchymal phase, the PMD in the tissue increases further.

The PMD values of different phases of enhanced CT vary greatly. Jeroen et al. [25] believed that the images in venous phase should be used for analysis. They believed that the tissue contrast of venous phase images was clearer; therefore, the data measured by software were more accurate. However, our findings differ. Our results showed that ΔPMD increased faster in patients with low PMD and slower in patients with normal PMD, both in the arterial, venous, and parenchymal phases (similar to the findings of Boutin et al. [31]), resulting in reduced differences between patients' intrinsic PMD after CT enhancement, which is not conducive to reflecting differences between patients. We further explored the reasons for the change in ΔPMD. A previous study showed that the increase in CT density after enhancement was directly related to the body weight of patients [32]; therefore, we compared the differences in body weight, BMI, and psoas muscle areas representing muscle content of the population in different PMD groups and found that the differences in body weight and psoas muscle areas between the low PMD group and the normal PMD group were statistically significant, patients with low PMD had a small body weight, and bolus injection of the same dose of contrast agent increased the contrast agent content per unit muscle of patients, resulting in a difference in ΔPMD. Therefore, we concluded that patients in the low PMD group had higher ΔPMD after enhancement than patients in the normal PMD group, and the change made the difference in PMD between different individuals smaller; therefore, it is not recommended to use enhanced CT to assess the PMD status of patients.

Our study has several limitations. First, this is a retrospective study, we could not obtain the data of cardiac function of the patients, and the cardiac function status has an effect on the distribution of contrast agent, which needs to be compensated for in the future prospective study. Meanwhile, the contrast agent used in enhanced CT in this study was a fixed dose administration method; therefore, the body weight and psoas muscle areas had a greater impact on PMD, and if the contrast agent is administered according to body weight, the change of PMD after enhancement requires further study. Moreover, our results may have limited generalizability to other patient cohorts, which needs further investigation and verification.

In conclusion, for the study with PMI and PMD as indicators, different stages of enhanced CT for fixed dose administration have no effect on the PMI values of manual measurement but can change the results of PMD. This change makes the difference of original information smaller and the cost higher. Therefore, it is not recommended to use enhanced CT routinely with fixed dose administration of contrast agent for PMI and PMD assessment.

## Figures and Tables

**Table 1:** Characteristics of patients.

Demographics	All (*N* = 45)
Age in years	61.69 ± 10.36 (39–80)
Weight, kg	65.19 ± 13.01
Height, m	1.66 ± 0.08
Sex, male: female	26 : 19
Body mass index, kg/m^2^	23.56 ± 3.42

**Table 2:** Differences in psoas muscle areas and PMI measurements using contrast-enhancement CT.

	PMI			Psoas muscle areas		
	Mean ± SD	*F* value	*p* value	Mean ± SD	*F* value	*p* value
Unenhanced phase	6.905 ± 2.170	2.206	0.090	19.328 ± 7.003	2.256	0.085
Arterial phase	6.886 ± 2.195			19.280 ± 7.060		
Portal-venous phase	6.923 ± 2.239			19.384 ± 7.169		
Delayed phase	6.866 ± 2.218			19.223 ± 7.106		

**Table 3:** Differences in PMI classification using contrast-enhancement CT (*n* = 45).

	PMI (low)	PMI (normal)	*X* ^2^ value	*p* value
Unenhanced phase	8	37	0.667	0.881
Arterial phase	9	36		
Portal-venous phase	10	35		
Delayed phase	11	34		

**Table 4:** Differences in PMD measurements using contrast-enhancement CT.

	PMD (mean ± SD)	*F* value	*p* value
Unenhanced phase	34.311 ± 7.535	188.046	*p* < 0.001
Arterial phase	37.478 ± 7.118		
Portal-venous phase	40.689 ± 7.116		
Delayed phase	42.989 ± 7.745		

**Table 5:** Comparison of PMD values between PMD low group and PMD normal group at different phases of CT.

	PMD low group (mean ± SD)	PMD normal group (mean ± SD)	*T* value	*p* value
PMD in unenhanced phase	31.032 ± 6.136	41.571 ± 4.783	−5.682	*p* < 0.001
PMD in arterial phase	34.516 ± 5.995	44.036 ± 4.618	−5.266	*p* < 0.001
PMD in portal-venous phase	38.081 ± 6.305	46.464 ± 5.246	−4.336	*p* < 0.001
PMD in delayed phase	40.613 ± 7.290	48.250 ± 6.091	−3.413	0.001

**Table 6:** Differences in ΔPMD using contrast-enhancement CT.

	PMD low group (median)	PMD normal group (median)	*T* value	*p* value
ΔPMD (arterial phase)	3.484 ± 2.227	2.464 ± 2.656	1.339	0.188
ΔPMD (portal-venous phase)	7.048 ± 3.067	4.893 ± 2.558	2.289	0.027
ΔPMD (delayed phase)	9.581 ± 3.033	6.679 ± 2.621	3.092	0.003

**Table 7:** Comparison of body weight, BMI, and psoas muscle areas between low PMD group and normal PMD group.

	PMD low group (mean ± SD)	PMD normal group (mean ± SD)	*T* value	*p* value
Body weight	61.484 ± 10.769	73.393 ± 14.148	−3.110	0.003
BMI	22.903 ± 3.199	25.000 ± 3.565	−1.965	0.056
Psoas muscle areas	16.142 ± 5.259	26.382 ± 4.937	−6.158	*p* < 0.001

## Data Availability

The raw data supporting the conclusions of this article will be available from the authors, without undue reservation.
